# Human lymph node degeneration in the thoracic region: A morphometric and immunohistochemical analysis using surgically obtained specimens

**DOI:** 10.3389/fphys.2022.990801

**Published:** 2022-09-14

**Authors:** Zhe-Wu Jin, Masaya Aoki, Kazuhiro Ueda, Go Kamimura, Aya Takeda-Harada, Gen Murakami, Masami Sato

**Affiliations:** ^1^ Department of Anatomy, Wuxi School of Medicine, Jiangnan University, Wuxi, China; ^2^ Department of General Thoracic Surgery, Kagoshima University School of Medicine, Kagoshima, Japan; ^3^ Department of Anatomy, Tokyo Dental College, Tokyo, Japan

**Keywords:** lymph node hyalinization, cortical degeneration, medullary sinus, fibrosis, lipomatosis

## Abstract

Lymph node degeneration was examined in 539 mediastinal and intrapulmonary nodes removed from 78 patients, aged 49–82 years, without cancer metastasis. Medullary sinus hyalinization observed in 36.2% of the hilar and 38.5% of the interlobar nodes. Early and smaller lesions were eosinophilic and factor VIII-positive, whereas advanced and large lesions contained a bulky mass of collagenous fiber bundles with few slender cells positive for smooth muscle actin (SMA) and factor VIII, as well as anthracotic macrophages. Subcapsular sinus hyalinization, observed in 4.3% of hilar nodes, was detected as a thick fibrous layer (over 0.2 mm) between the surface cortex and the thickened capsule. The fibrous layer contained SMA-positive slender cells, whereas the thickened capsule contained fibers positive for elastin and factor VIII. These hyalinization lesions occupied 3.6% and 0.8% of the sectional areas of hilar and lower paratracheal nodes, respectively. Areas of early and small cortical degeneration, surrounded by fibers positive for SMA and vimentin, did not contain lymphocytes and macrophages, but contained abundant small stromal cells. Silver staining suggested that advanced cortical degeneration was composed of collagen fibrils other than type I. Fatty tissues, seen in 47.8% of hilar nodes, often extended into and replaced medullary sinus tissue. Island-like remnants of medullary sinuses in areas of fatty degeneration contained various stromal cells positive for SMA, elastin, factor VIII and/or CD34. These degenerative morphologies, however, did not correlate with either age or smoking index. The present cortical degeneration usually seemed to follow hyalinization, but both were likely to occur independently.

## Introduction

Lymph node hyalinization, defined as an onion-peel or amorphous, eosinophilic lesion comprised of non-branching collagen fibrils (Taniguchi et al., 2003), may be a major aspect of degeneration, although descriptions in textbooks remain unclear (e.g., van der Valk and Meijer, 1997). Axillary node hyalinization increases with age (Tsakraklides et al., 1975) and tends to be associated with breast cancer metastasis (Anastassiades and Prycem, 1966). This hyalinization, however, has not been reported in experimental animals, even in aged animals (Hoshi et al., 1986). Mediastinal nodes are characterized by large hyalinization lesions in medullary sinuses, whereas other nodes, especially pelvic nodes, are characterized by small hyalinization lesions in the follicles (Taniguchi et al., 2003). Composite collagen fibrils in areas of pelvic-type hyalinization are three times thicker (150 nm) than fibrils in areas of mediastinal node hyalinization (Taniguchi et al., 2003).

Morphologically, areas of hyalinization consist of 1) an onion peel-like arrangement of slender cells replacing a follicle in the cortex; and 2) an amorphous eosinophilic matrix and/or irregularly arrayed thick collagen bundles occupying the nodal sinuses (Taniguchi et al., 2003). Although that study regarded these two morphologies as a single entity, the study focus was not the degenerative morphology itself but its site-dependent differences. Moreover, that study did not include immunohistochemical evaluations, possibly because of the poor fixation of donated cadavers. The present study hypothesized that early stage morphology would differ in sinus hyalinization and cortical degeneration. Hadamitzky et al. (2010) performed an excellent semi-quantitative study of the inguinal node degeneration, but they did not discriminate fibrotic lesions between the sinuses and cortex of the node.

Recently, Erofeeva and Mnikhovich (2018, 2020) reviewed their many studies on age-dependent fibrosis of human mediastinal nodes. They evaluated the degree of fibrosis according to numbers of composite cells such as fibroblasts and reticular cells. Thus, they hypothesized that greater contents of these cells indicate the advanced fibrosis. However, the site and type of fibrous tissues primarily determine the cell density: like a ligament or tendon, even a bulky hyalinization was likely to contain few cells. Moreover, they did not evaluate the nodal fibrosis using immunohistochemistry of cells possibly responsible for the fiber production. Consequently, using 539 thoracic nodes removed from 78 patients without cancer metastasis, the aim of the present study was to compare the histology, immunohistochemistry, morphometry, and etiology of these two types of nodal degeneration in surgically obtained specimens.

## Materials and methods

This study included 539 nodes that had been surgically removed from 78 patients suffering from lung cancer, mean age 69.1 years (range, 49–82 years), without cancer metastasis. The 78 patients included 48 men, of mean age 68.9 years, and 30 women, of mean age 69.3 years. Of these 78 patients, 37 (47.3%) had smoked for >20 years before surgery. The cancer cell types were adenocarcinoma (51 patients; 25 men, and 26 women), squamous cell carcinoma (15; 14 and 1) and other types such as neuroendocrine and pleomorphic carcinomas (12; 9 and 3). Any presurgical anticancer treatment, such as radiation and chemotherapy, was not performed for these patients. Of the 539 lymph nodes, 172 were lower paratracheal, 10 were subaortic, 127 were subcarinal, 69 were hilar, and 161 were interlobar. When limited to these five sites examined, numbers of nodes obtained from each patient ranged from 2 to 18: they included various size of nodes. Likewise, the size of nodes was quite different between patients (see the final subsection of the Results).

The removed nodes had been fixed in 10% w/w neutral formalin solution for 7 days, followed by routine paraffin-embedding for histologic examination. About 15–20 serial sections were prepared from each node, including the maximum sectional area of the node. Sections were stained with hematoxylin and eosin (HE), silver stain, and specific antibodies. Silver staining, usually called “Gitter (reticular network) staining”, was performed as described previously (Lillie et al., 1980); this method differed from both Gomori’s method (e.g., Satoh et al., 1997) and PAM staining (Yamanouchi, 1969). The use of the surgical specimens was approved by the Ethics Committee of Kagoshima University Graduate School of Medicine and Dentistry (No. 210198).

### Immunohistochemistry

The primary antibodies, along with their dilutions and antigen retrieval procedures, are shown in Table 1. Briefly, antibodies were directed against the B-lymphocyte markers IgM and CD79a, the T lymphocyte markers CD4 and CD8, the macrophage marker CD68 , the proliferating cell marker Ki67, the lymphatic vessel marker D2-40 (podoplanin), the blood vessel endothelium markers factor VIII and CD34, the elastic and oxytalan fiber marker alpha elastin, the Langerhans cell marker S100, and the stromal cell and fiber markers smooth muscle actin (SMA) and vimentin. After incubation with the primary antibodies, the tissue specimens were incubated for 30 min with a 1:1,000 dilution of horseradish peroxidase (HRP) conjugated secondary antibody (Histofine Simple Stain Max-PO, Nichirei, Tokyo), and antigen-antibody reactions were detected by incubation for 3–5 min with diaminobenzidine (Histofine Simple Stain DAB, Nichirei). The antigen-antibody reactions were detected by the HRP-catalyzed reaction with diaminobenzidine and this DAB reaction made the positive cells or fibers brown. Counterstaining with hematoxylin was performed on the same samples. Negative controls consisted of tissue samples without the primary antibody. Arterial walls and lymphatic vessels outside nodes in the same section were used as positive controls for SMA, elastin and D2-40 respectively. All samples were counterstained with hematoxylin. In addition, D2-40 is known as a marker of follicular dendritic cells in lymph nodes (Yu et al., 2007; Marsee et al., 2009).

**TABLE 1:** Primary antibodies and their dilution and specific treatment.

Ig M	Rabbit poly	Dako N1509 (Grostrup) 1:100, protease
CD79α	Mouse mono	Dako IR621 (Grostrup) ready-to-use, autoclave (121°C, 5 min)
CD4	Mouse mono	Nichirei 413181 (Tokyo) 1:80, autoclave (121°C, 5 min)
CD8	Mouse mono	Nichirei 413211 (Tokyo) 1:50, autoclave (121°, 5 min)
CD68	Mouse mono	Dako N0814 (Grostrup) 1:200, trypsin
Ki-67	Mouse mono	Dako M7240 (Grostrup) ready-to-use
Factor VIII	Rabbit poly	Dako GA527 (Grostrup) 1:16000, trypsin
CD34	Mouse mono	Nichirei 413361 (Tokyo) 1:200
*α* smooth muscle actin	Mouse mono	Dako M0851 (Grostrup) 1:800, trypsin
*α* elastin	Mouse mono	Abcam ab9519 (Cambridge) 1:20, trypsin
Vimentin	Mouse mono	Dako IR630 (Grostrup) ready-to-use, microwave
D2-40 (podoplanin)	Mouse mono	Signet (Dedham), 1:200, microwave
S 100 protein	Mouse mono	Dako Z0311 (Grostrup) 1:100

Mono, monoclonal; poly, polyclonal; Grostrup in Denmark; Dedham in MA United States.

The anti-SMA antibody used in the present study has been reported to stain the endothelium of arteries and veins as well as smooth muscle, but does not react with lymphatic endothelium (Hayashi et al., 2008). Moreover, vimentin, which is present in one type of intermediate filament, coexists with desmin in developing muscle fibers and plays an important role in maintaining the stability of cells and signal transduction (Yang and Makita, 1996; Abe et al., 2010). Vimentin is also expressed in the cell processes of osteoblasts and osteocytes (Shapiro et al., 1995). Thus, positive staining with anti-vimentin antibody suggests that the tissue or cell is under mechanical stress (Abe et al., 2010; 2011a). CD34 is present not only in vessels but in stromal cells and fibers of many organs and tissues (Abe et al., 2011b). Benign sinus histiocytosis is frequently observed in lymph nodes, with this pathology characterized by an increased number of Langerhans cells, which are positive for S100 (van der Valk and Meijer, 1997).

### Morphometric analysis

The maximum sectional area of each HE-stained node, as well as the proportional areas represented by hyalinized and pre-hyalinized lesions, were measured. After manual tracing of the node and lesions, the scanned images (Adobe Photoshop^®^ images) of each site under ×1 objective lens were processed using ImageJ version 1.45 software (U.S. National Institutes of Health). All statistical analyses were performed with the Statistical Package for the Social Sciences (SPSS version 26.0). The correlations between continuous variables such as proportional areas of hyalinized and pre-hyalinized lesions, smoking index (numbers of cigarettes per day x years of past smoking) and age were analyzed by Spearman’s test. A *p-*value of <0.05 was considered statistically significant.

### Photographs

Most photographs for histology were taken with a Nikon Eclipse 80, whereas photographs at ultra-low magnification (objective lens less than ×1) were obtained using a high-grade flat scanner with translucent illumination (Epson scanner GTX970).

## Results

### Usual fibrous componennts and areas of pre-hyalinization

**FIGURE 1:**
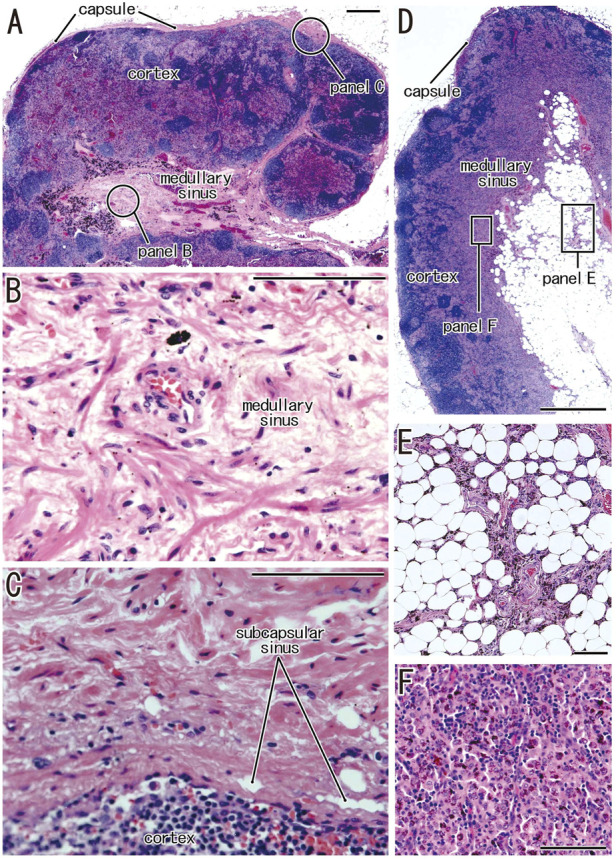
Increased collagenous bundles in the subcapsular and medullary sinuses and the fatty degeneration in the medullary sinus. HE staining. **(A–C)** display a hilar node removed from a 78-years-old man (smoker); **(D,E)**, a right lower paratracheal node from a 63-years-old man (smoker). **(B,C)** are higher magnification views at sites indicated by circles in **(A)**, respectively. Thick collagenous bundles are increased in the subcapsular and medullary sinuses [their usual morphologies, see **(F)** and Figures 2, 3]. The fatty tissue extends into the medullary sinus **(D)** and a fragment of the sinus tissue is surrounded by fatty tissues **(E)**. **(F)** exhibits the usual medullary sinus with high cellularity and without thick collagen bundles. Scale bars: 1 mm in **(A,D)**; 0.1 mm in **(B,C,E,F)**.

Clusters of thick collagenous fiber bundles were often present in small or large areas of medullary sinuses (Figures 1A,B). This medullary sinus fibrosis was observed at one or multiple nodes removed from a single lower paratracheal, subaortic, subcarinal, hilar or interlobar site. Because of the thick collagenous bundles, the usual high cellularity was lost in the medullary sinuses (Figures 1B,F). Similarly, some nodal capsules became hypertrophic, with subcapsular sinuses being embedded in collagenous tissue (Figure 1C). These fibrous lesions are called pre-hyalinized sinuses. The pre-hyalinized areas occupied 4.6%–5.4% of the sectional areas of the lower paratracheal, subcarinal, hilar, and interlobar nodes (Table 2). In addition, fatty tissues at the hilum of the nodes often extended into the medullary sinus to replace the medullary tissue (Figures 1D,E).

**TABLE 2:** Hyalinization and pre-hyalinization in thoracic nodes.

Site (numbers)	Medullary hyalinization	Subcapsular hyalinization	Hyalinization % area ± SD^b^	Pre-hyalinization % area ± SD^b^
Lower PT^a^	22/172 nodes	3	29/3,688 mm^2^	200/3,688 mm^2^
	(12.8%)	(1.7%)	(0.8% ± 5.7%)	(5.4% ± 3.7%)
Sabaortic	2/10 nodes	1	13/305 mm^2^	22/305 mm^2^
	(20.0%)	(10.0%)	(4.3% ± 4.9%)	(7.2% ± 6.2%)
Sabcarnial	27/127 nodes	4	88/4,040 mm^2^	194/4,040 mm^2^
	(21.3%)	(3.1%)	(2.2% ± 6.4%)	(4.8% ± 3.5%)
Hilar	25/69 nodes	3	74/2,184 mm^2^	118/2,184 mm^2^
	(36.2%)	(4.3%)	(3.4% ± 11.3%)	(5.4% ± 5.0%)
Interlobar	62/161 nodes	6	156/4,326 mm^2^	199/4,326 mm^2^
	(38.5%)	(3.7%)	(3.6% ± 9.0%)	(4.6% ± 3.1%)

a PT, paratracheal node.

b %area, a proportional area of the medullary and subcapsular hyalinization or pre-hyalinization in the maximum sectional area of the node.

**FIGURE 2:**
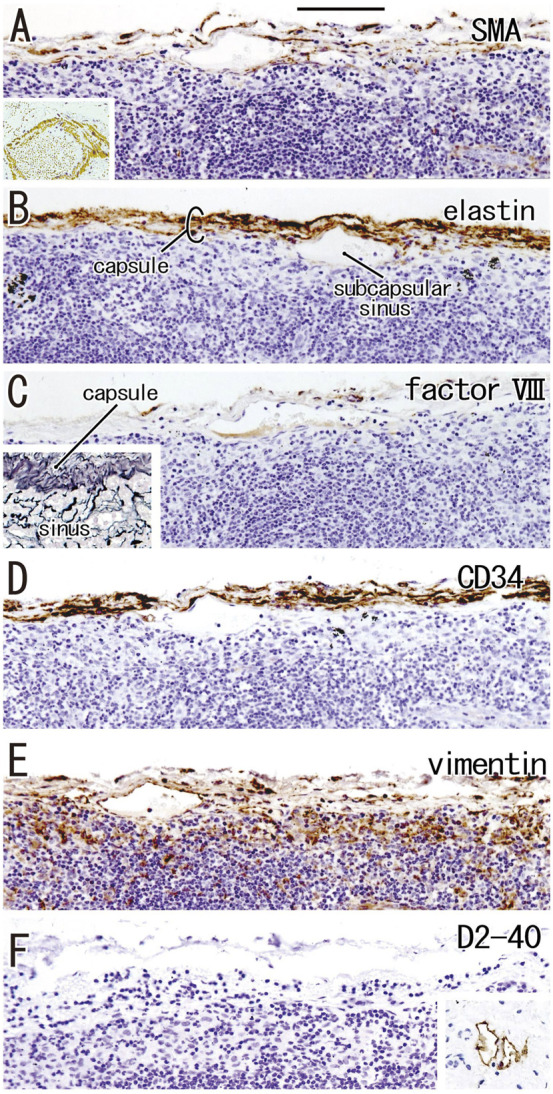
Immunohistochemistry of the lymph node capsule and subcapsular sinus: the usual morphologies. A subcarinal node removed from a 63-years-old woman (non-smoker). The DAB reaction made the positive cells brown. The nodal capsule expresses strong reactivity of elastin and CD34 **(B,D)** and it contains slender cells positive for immunostainings of smooth muscle actin (SMA) and vimentin **(A,E)**. An insert at the lower angle of **(A)** shows a positive control of the present SMA antibody (artery found in the same section). Few cells express factor VIII **(C)**. An insert at the lower angle of **(C)** displays silver staining of a near section containing violet colored thick fibers in the capsule and blue-gray colored reticular fibers in the subcapsular sinus. No reactivity is seen for D2-40 [podoplanin; **(F)**] in contrast to lymph vessels around the node [insert at the lower angle of **(F)**]. All panels, including an insert in **(F)**, were prepared at the same magnification [scale bar in **(A)**, 0.1 mm].

**FIGURE 3:**
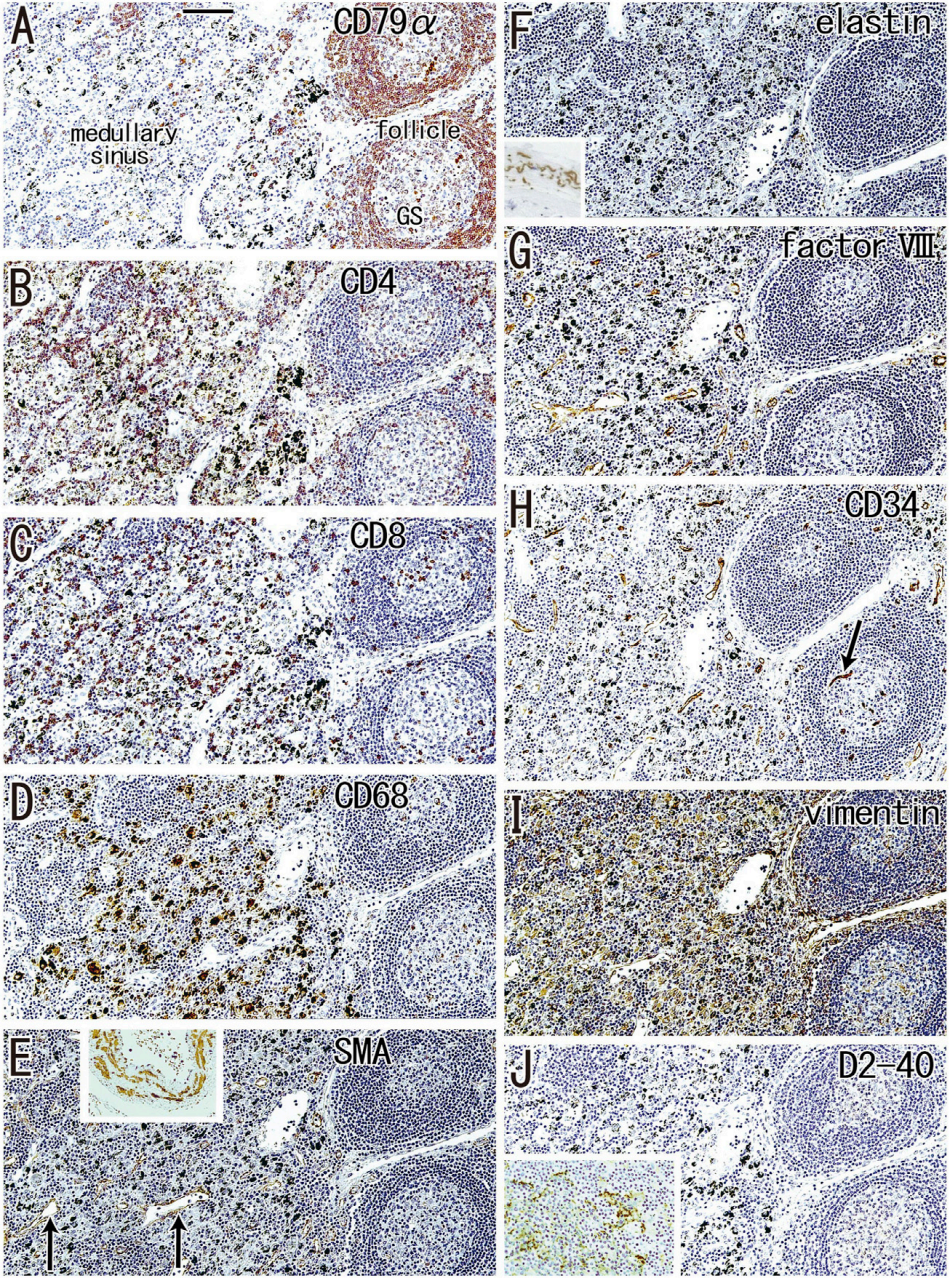
Immunohistochemistry of the lymph follicle and medullary sinus: the usual morphologies. An interlobar node removed from a 64-years-old woman (non-smoker). The DAB reaction made the positive cells brown. CD79a-positive B lymphocytes surrounds germinal center (GS) in **(A)**. The medullary sinus contains abundant CD4^−^or CD8-positive T lymphocytes **(B,C)**. In the medullary sinus, almost all of CD68-positive macrophages carry carbon particles [anthracotic macrophages; **(D)**]. Endothelium of thin arteries [arrows in **(E)**] is positive for smooth muscle actin [SMA; **(E)**]. An insert at the upper margin of **(E)** shows a positive control of the present SMA antibody (artery found in the same section). Factor VIII-positive vessels are absent in germinal center **(G)** in contrast to CD34-positive vessel [arrow in **(H)**]. Vimentin reactivity **(I)** appears to be present in both macrophages and some stromal cells in the medullary cells. No reactivity is seen for either elastin **(F)** or D2-40 [podoplanin; **(J)**]. An insert at the lower angle of **(F)** exhibits the positive control of elastin immunostaining (an arterial wall outside of the node). An insert at the lower angle of **(J)** displays D2-40-positive follicular dendritic cells in another specimen (a 70-years-old man, smoker). All panels were prepared at the same magnification [scale bar in **(A)**, 0.1 mm].

According to immunohistochemical analysis of lymph nodes without hyalinization (Figures 2, 3), nodal capsules were strongly positive for elastin and CD34 (Figures 2B,D), with slender cells in these capsules being positive for SMA and vimentin (Figures 2A,E). Few cells in the capsules were positive for factor VIII (Figure 2C), whereas the sinus endothelium was consistently negative for D2-40 (podoplanin) (Figures 2F, 3J). Few anthracotic macrophages were present in the subcapsular sinuses. Blood vessel endothelium in the medullary sinuses was positive for factor VIII, CD34 (Figures 3G,H) and SMA (Figure 3E). In contrast to factor VIII, CD34 immunostaining showed the presence of thin blood vessels in the follicles and germinal centers. Elastin was absent from the thin vessels in the medullary sinuses (Figure 3F). Both macrophages and stromal cells in the medullary sinuses were positive for vimentin (Figure 3I).

Although D2-40 is known as a marker of follicular dendritic cells (see the Materials and Methods), the present specimens usually exhibit very weak or negative reactivity possibly because of the long and deep fixation. However, in some specimens, the follicular dendritic cells were well recognized in the staining (insert in Figure 3J).

### Hyalinization of the medullary and subcapsular sinuses

**FIGURE 4:**
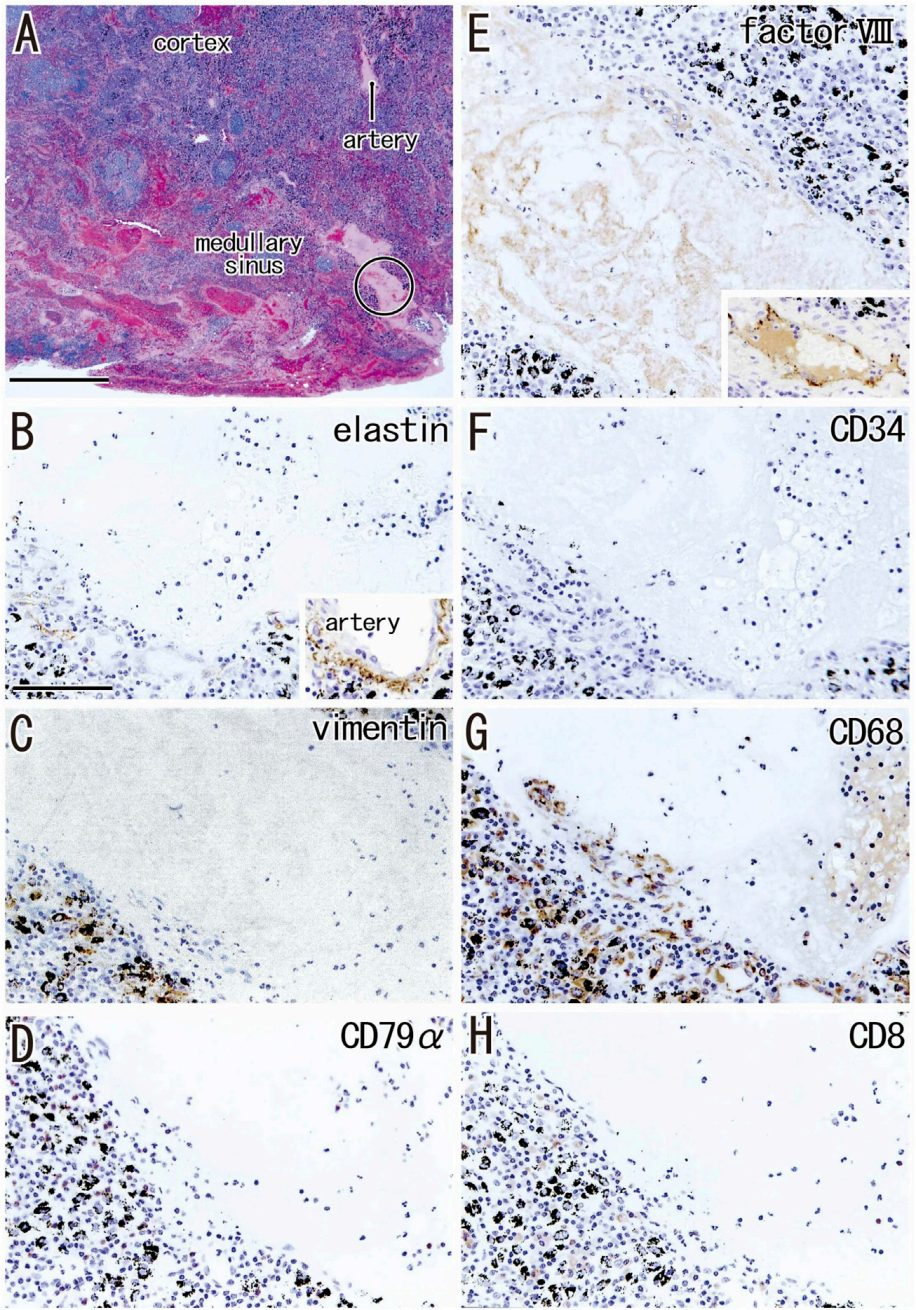
Early phase of the medullary sinus hyalinization. A subaortic node removed from a 70-years-old man (smoker). HE staining **(A)** and immunohistochemistry [**(B–H)**; near sections of **(A)**]. **(B–H)** are higher magnification views of a site indicated by a circle in panel **(A)**. The DAB reaction made the positive cells brown. The hyalinization, negative for immunostaining of elastin and CD34 **(B,F)**, contains no or few lymphocytes expressing CD79a or CD8 **(D,H)**. An insert at the lower angle of **(B)** exhibits the positive control of elastin immunostaining (an artery outside of the node). Vimentin-positive cells in the hyalinization **(C)** appear not to be CD68-positive **(G)**. Abundant macrophages carry carbon particles (anthracotic macrophages). A weak reactivity of factor VIII **(E)** is similar to a staining of postmortem hemolysis in a thin vein [insert at the lower angle of **(E)**]. **(B–H)** were prepared at the same magnification [scale bars: 1 mm in **(A)**; 0.1 mm in **(B)**].

Hyalinization of the medullary sinuses was observed in 36.2% of the hilar nodes and 38.5% of the interlobar nodes, but in only 12.8% of the paratracheal nodes (Table 2). Medullary lesions were larger and more frequently present in the intrapulmonary hilar and interlobar nodes than in the mediastinal nodes, constituting 3.6% of the proportional area of interlobar nodes compared with 0.8% of the proportional area of lower paratracheal nodes (Table 2). Small and slender-shaped lesions at the margins of the medullary sinuses appeared amorphous and even soft (Figure 4A), possibly because the composite fibers were thinner and less dense. This morphology indicated an early phase of hyalinization. Areas of early hyalinization were negative for elastin and CD34 (Figures 4B,F) and contained no or few lymphocytes (Figures 4D,H). Vimentin-positive cells in areas of hyalinization (Figure 4C) were unlikely to be macrophages (Figure 4G). Early hyalinization was characterized by a matrix weakly positive for factor VIII (Figure 4E), with a color similar to that of homogeneous staining of hematolysis in a blood vessel (Figure 4E, insert).

**FIGURE 5:**
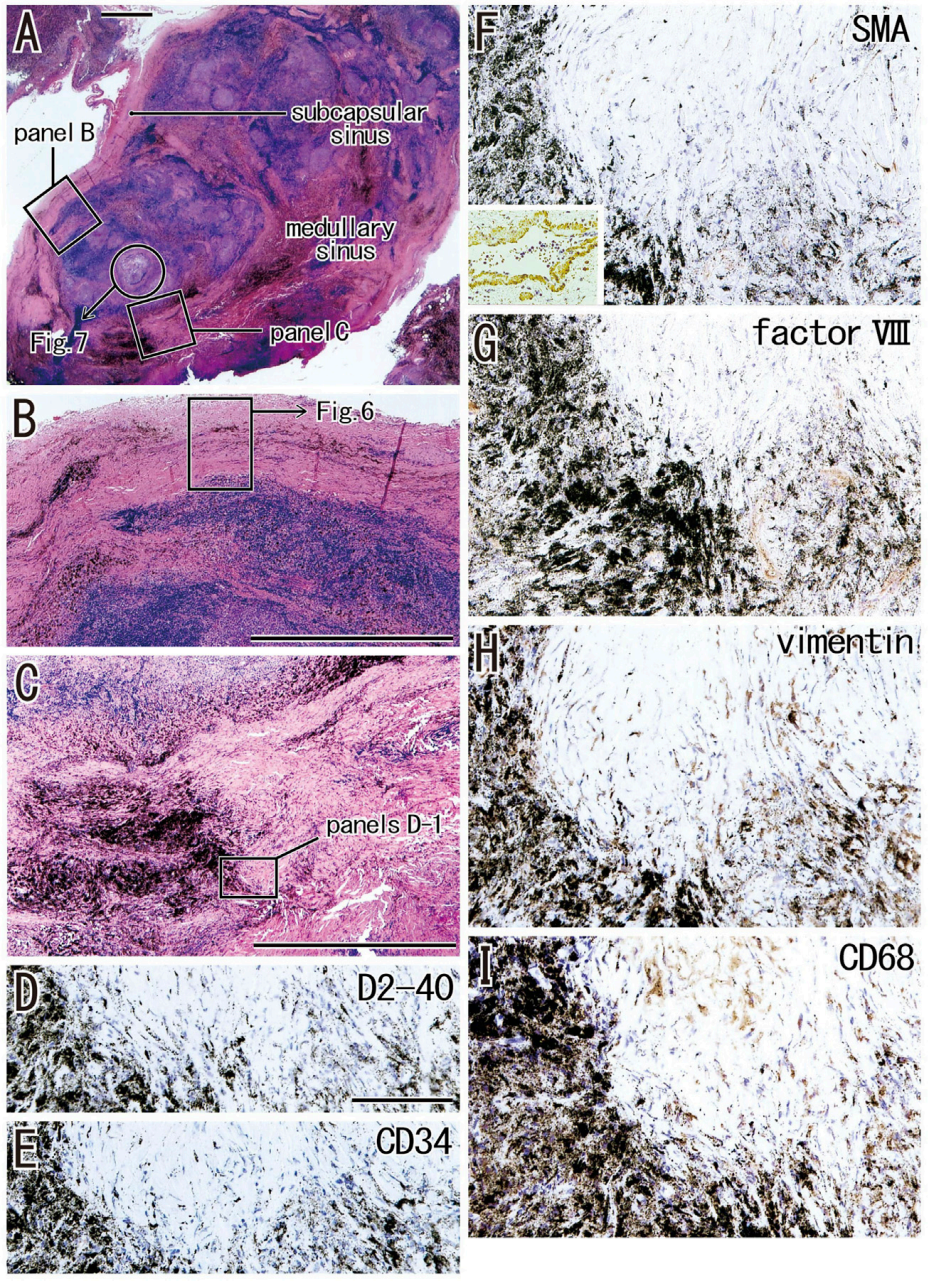
Advanced sinus hyalinization in combination with the cortex degeneration. An interlobar node removed from a 78-years-old man (smoker). Two squares in **(A)** are shown at the higher magnification in **(B)** (subcapsular sinus hyalinization; immunohistochemistry, see Figure 6) and **(C)** (medullary sinus hyalinization). A part of the cortex [circle in **(A)**] will be shown in Figure 7 with immunohistochemistry. The DAB reaction made the positive cells brown. The medullary sinus hyalinization, negative for D2-40 [podoplanin; **(D)**] and CD34 **(E)**, contains few slender cells positive for smooth muscle actin [SMA; **(F)**], factor VIII **(G)**, vimentin **(H)** or CD68 **(I)**. An insert at the lower angle of **(F)** shows a positive control of the present SMA antibody (artery found in the same section). Abundant macrophages carry carbon particles (anthracotic macrophages) and some of them are very large. Some of vimentin-positive cells appear to be CD68-positive macrophages. **(B,C)** or **(D–I)** were prepared at the same magnification [scale bars; 1 mm in **(A–C)**; 0.1 mm in **(D)**].

When areas of hyalinization occupied all or most of a medullary sinus (Figures 5A,C), the lesion appeared hard because of the presence of irregularly arrayed bulky fibers. These lesions often contained abundant anthracotic macrophages, with the bulky fibers converting large round clusters of anthracotic macrophages to a flat sheet or fragments. Areas of bulky hyalinization, which were negative for D2-40 (podoplanin) and CD34 (Figures 5D,E), contained non-anthracotic macrophages (Figure 5I) and other fibrous cells positive for SMA (Figure 5F), factor VIII (Figure 5G) and/or vimentin (Figure 5H). Some vimentin-positive cells appeared to be positive for CD68.

**FIGURE 6:**
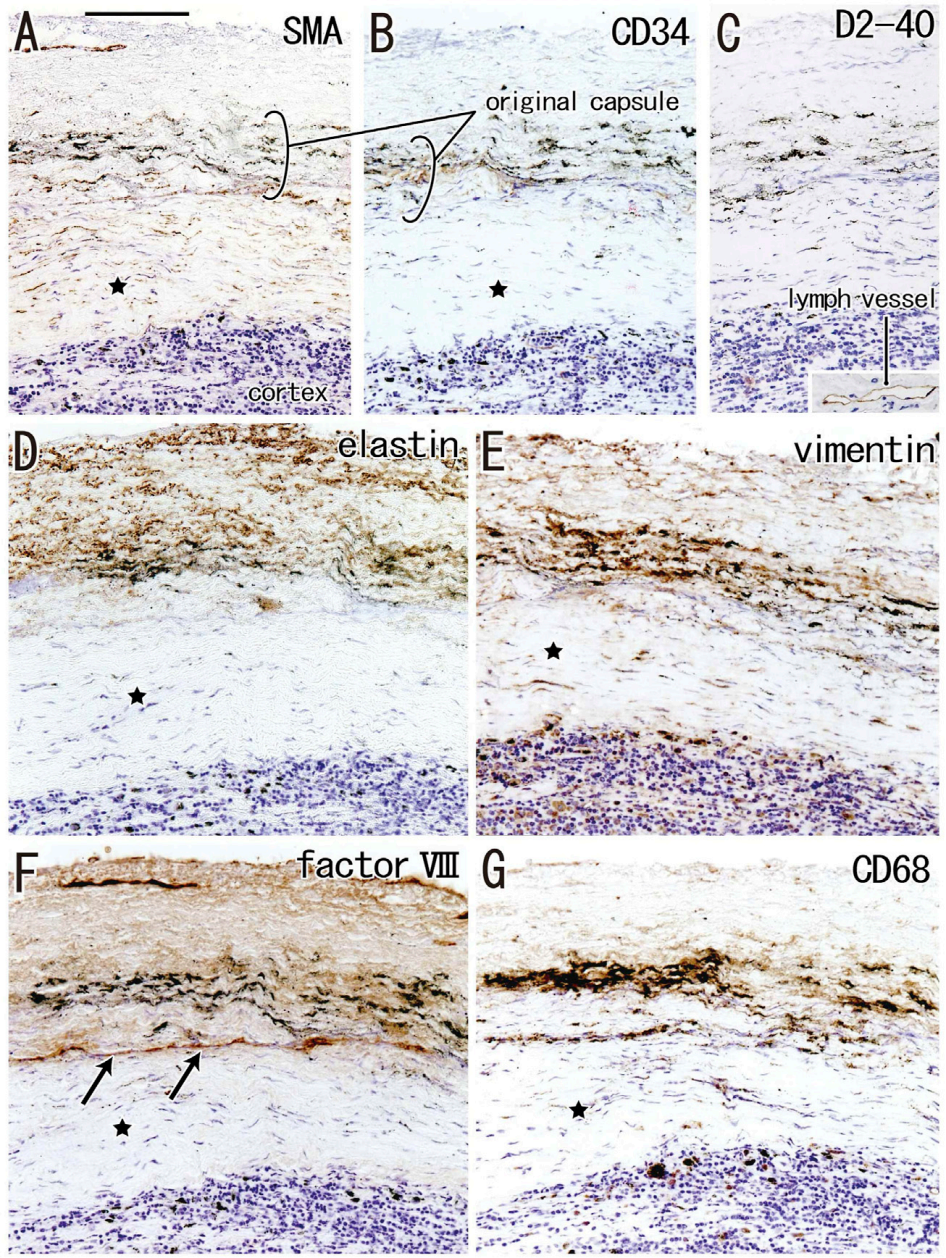
Immunohistochemistry of the subcapsular sinus hyalinization. Near sections of Figure 5A and higher magnification views of a site indicated by a square in Figure 5B (an interlobar node removed from a 78-years-old man). The DAB reaction made the positive cells brown. According to immunostainings of smooth muscle actin (SMA) and CD34 **(A,B)**, the original nodal capsule is separated from the surface cortex by a thick fibrous zone (star). No reactivity is seen for D2-40 [podoplanin; **(C)]** except for lymph vessels around the node [insert at the bottom of **(C)**]. Abundant elastin-positive fibers **(D)** are seen in the outside of the original capsule (cf., Figure 2B). Vimentin reactivity is much stronger in the original capsule than the usual morphology (cf., Figure 2E). The fibrous zone (star) contains slender cells positive for SMA **(A)** or vimentin **(E)**. Conversely, the fibrous zone is negative for elastin, factor VIII or CD34. Notably, a factor VIII-positive fiber bundle delimits the fibrous zone [arrows in **(F)**]. No or few macrophages are seen in the zone **(G)**. Slender macrophages carry carbon particles (anthracotic macrophages). All panels, including an insert in **(C)**, were prepared at the same magnification [scale bar in **(A)**, 0.1 mm].

The incidence of subcapsular sinus hyalinization was much lower than the incidence of medullary hyalinization (Table 2). An advanced lesion with subcapsular sinus hyalinization was identified as a very thick “capsule” on HE staining (Figures 5A,B). However, rather than being a nodal capsule, the thickened structure was a “newly added” fibrous zone, based on the specific immunohistochemical features of the capsule (Figures 2, 6). Staining with antibodies to SMA and CD34 (Figures 6A,B) showed that the original nodal capsule was separated from the surface cortex by a thick fibrous zone of thickness ≥0.2 mm, in which thin fibers were arrayed in parallel. Notably, abundant elastin-positive fibers (Figure 6D) were present on the outside of the original capsule, thickening the capsule itself. Vimentin reactivity was much stronger in the original capsule than in the usual morphology (cf., Figure 2E). The fibrous zone contained slender cells positive for SMA or vimentin (Figures 6A,E). In contrast to medullary sinus hyalinization, this fibrous zone was negative for factor VIII, although a factor VIII-positive fiber bundle delimited the fibrous zone externally (Figure 6F). Subcapsular lesions consistently coexisted with medullary sinus lesions, although the latter were likely to be small. Notably, the proportional areas of hyalinized lesions per node did not correlate with either age (*p* = 0.846) or smoking index (*p* = 0.937).

### Cortical degeneration

**FIGURE 7:**
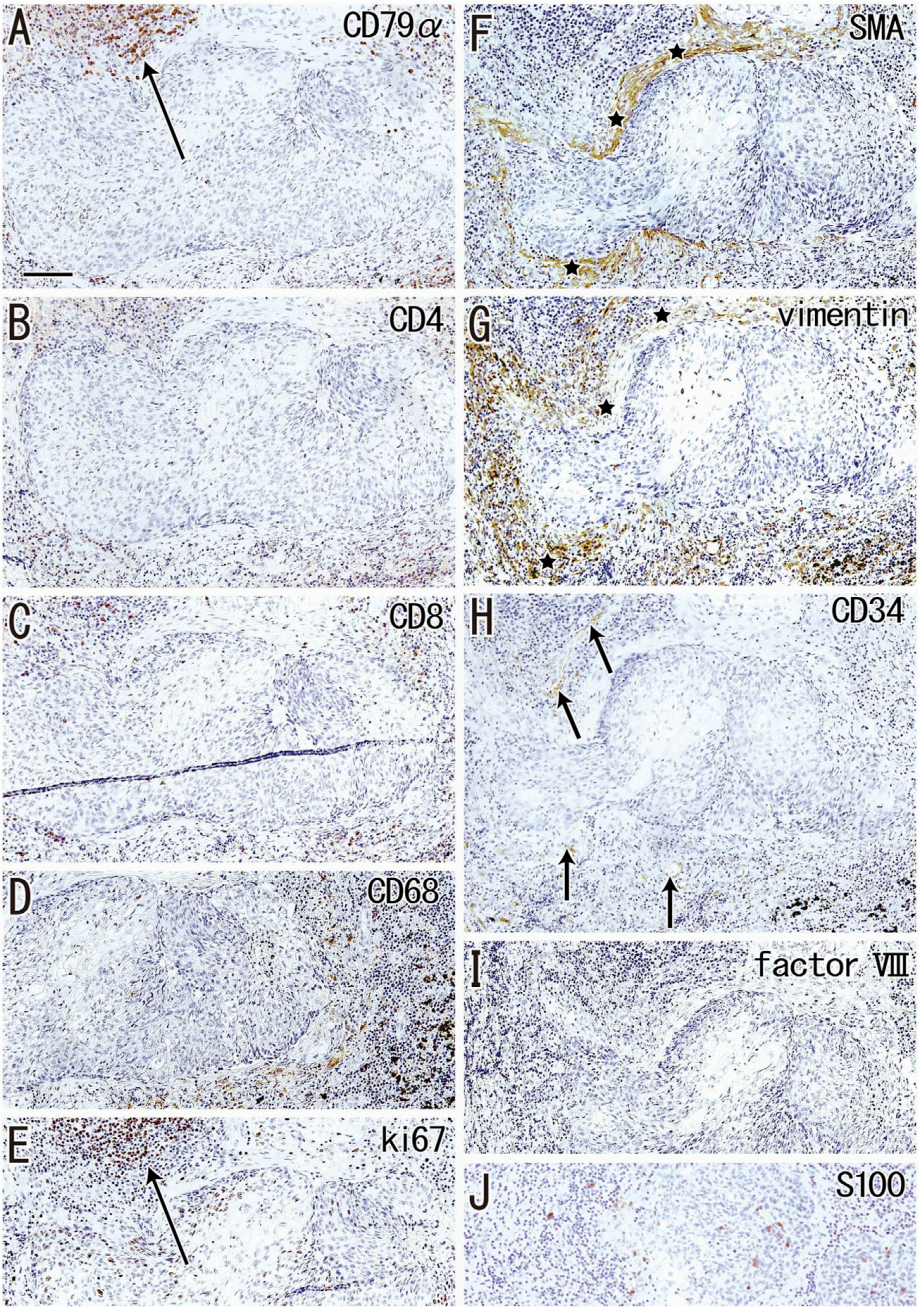
Immunohistochemistry of the cortical degeneration. Near sections of Figure 5A and higher magnification views of a site indicated by a circle in Figure 5A (an interlobar node removed from a 78-years-old man). The DAB reaction made the positive cells brown. A follicle-like structure (degenerative cortex) occupies in the center of each panel, but it contains no lymphocytes and macrophages positive for CD79a **(A)**, CD4 **(B)**, CD8 **(C)** or CD68 **(D)**. Ki67-positive proliferating cells (arrow in **(E)**) appear to be CD79a-positive B lymphocytes (arrow in **(A)**). The degenerative area is surrounded by fibers [stars in **(F,G)**] positive for smooth muscle actin (SMA) or vimentin. Factor VIII-positive vessels are absent **(I)**, but CD34-positive vessels [arrows in **(H)**] are present around the degenerative area. Langerhans’ cell candidates (S100 protein-positive; **(J)** are seen in and around the follicle-like structure. All panels were prepared at the same magnification [scale bar in **(A)**, 0.1 mm].

**FIGURE 8:**
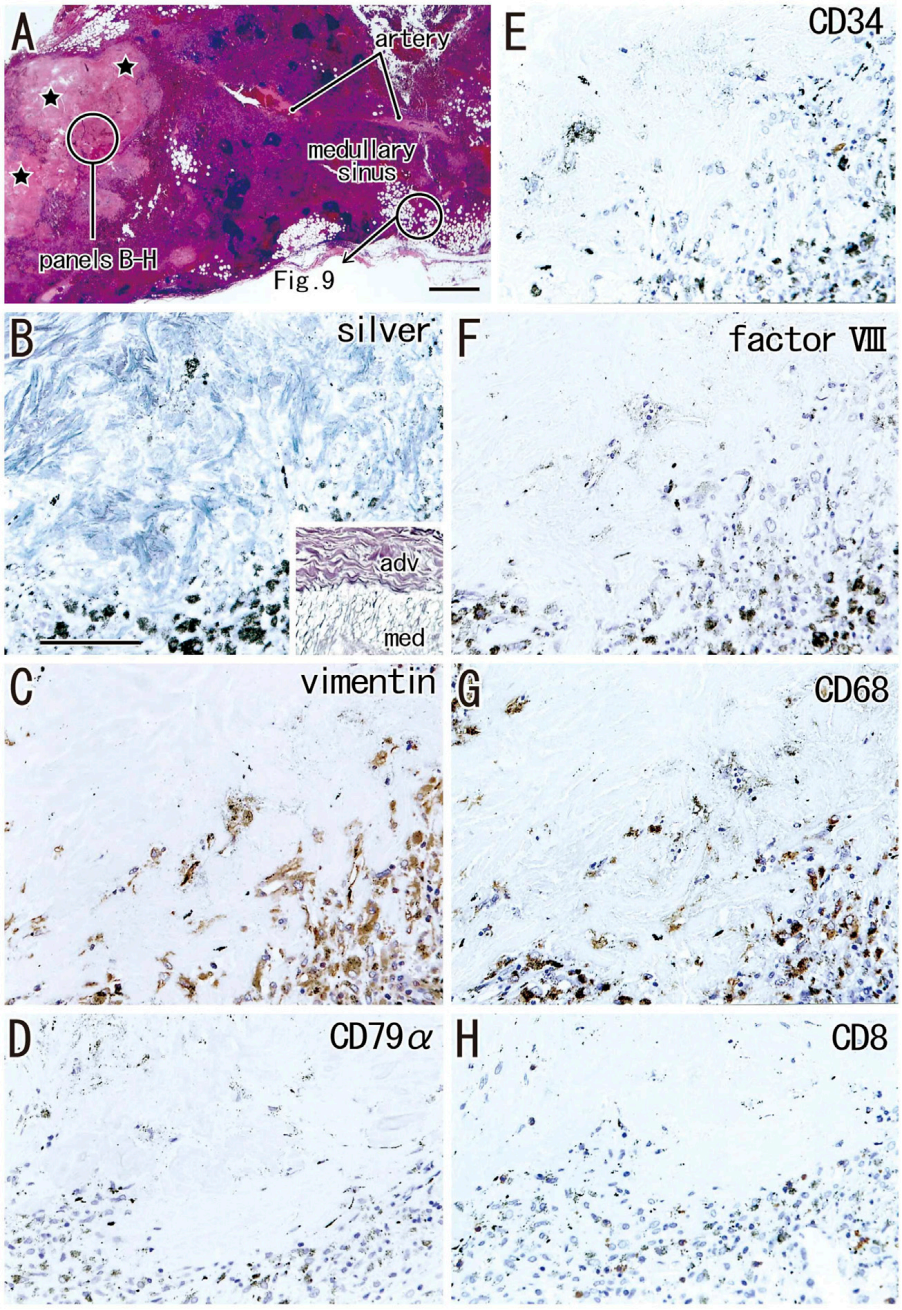
Immunohistochemistry of the cortical degeneration and the fatty degeneration. A hilar node removed from a 76-years-old man (non-smoker). This node contains a large degenerative area of the cortex [stars in **(A)**] and the fatty tissue extending into the medullary sinus. **(B–H)**, sections near **(A)**, display a site indicated by a circle in **(A)**. The former contains blue-gray colored thick fibers [silver staining; **(B)**] those are negative for elastin and smooth muscle actin (SMA; not shown). An insert at the lower angle of **(B)** exhibits silver staining of a wall of the artery outside of the node: type I collagen fibers of the adventitia (adv) stain red-brown, while basal laminae in the media (med) blue-gray. The degenerative area contains vimentin- or CD68-positive cells **(C,G)**. Anthracotic macrophages are black-colored. No cells in the area are positive for CD79a and CD8. CD34^−^or factor VIII-positive vessels are absent in the cortical degeneration. The DAB reaction made the positive cells brown. **(B–H)** were prepared at the same magnification [scale bars: 1 mm in **(A)**; 0.1 mm in **(B)**].

Irrespective of whether sinus hyalinization was evident in the node, some nodal cortices contained clusters of 1) large follicle-like structures different from the medullary cord (Figures 5A, 7) and/or 2) round-shaped masses comprised of bulky fibers (Figure 8). The incidence was much lower than that of sinus hyalinization, with the highest being 3.5% of lower paratracheal nodes (Table 3). The advanced morphology was somewhat similar to sinus hyalinization because of the inclusion of thick fiber bundles. Areas of cortical degeneration were proportionally larger in the lower paratracheal than in other nodes (Table 3).

**TABLE 3:** Cortical degeneration and fatty degeneration in thoracic nodes.

Site (numbers)	Fatty degeneration	Cortical degeneration	Cortical degeneration %area^b^ in the nodal section
Lower PT^a^	75/172 nodes (43.6%)	6 (3.5%)	6.7%–69.7% area of the node
Subaortic	1/10 nodes (10.0%)	0 (0.0%)	
Subcarinal	37/127 nodes (29.1%)	2 (1.6%)	2.6% or 9.1% area of the node
Hilar	33/69 nodes (47.8%)	3 (4.3%)	0.8%, 2.6% or 17.8% area of the node
Interlobar	52/161 nodes (32.3%)	3 (1.9%)	3.4%, 19.7% or 49.6% area of the node

a PT, paratracheal node.

b %area, a proportional area of the cortical degeneration in the maximum sectional area of the node.

Although the follicle-like structures (degenerative cortex) contained an abundance of small cells, these cells were unlikely to be lymphocytes or macrophages (Figures 7A–D). Lymphocytes surrounding these follicle-like structures were positive for the proliferation marker Ki67 (Figure 7E), although this applied only to lymphocytes in the germinal centers of usual nodes (not shown). Moreover, the degenerative area was surrounded by fiber bundles positive for SMA or vimentin (Figure 7F,G). Factor VIII-positive vessels were absent (Figure 7I), but CD34-positive vessels were present around the degenerative area (Figure 7H). Langerhans’ cells, positive for S100 protein, were seen in and around the cortical degeneration (Figure 7J), but the density varied significantly between patients.

Areas of advanced cortical degeneration did not contain small cells but did contain thick fiber bundles negative for elastin and SMA. Silver staining of these areas resulted in a blue-gray color similar to that resulting from silver staining of muscle cell basal lamina consisting of type IV collagen in contrast to the red-brown color of type I collagen fibers (Figure 8B). The degenerative area contained vimentin- and CD68-positive cells (Figures 8C,G). Vessels positive for CD34 and factor VIII were absent from the areas in and around advanced cortical degeneration (Figures 8E,F). Langerhans’ cells positive for S100 protein were absent from areas of cortical degeneration (not shown). The proportional areas of cortical degeneration per node did not correlate with either age (*p* = 0.964) or smoking index (*p* = 0.094).

### Fatty degeneration of the node

**FIGURE 9:**
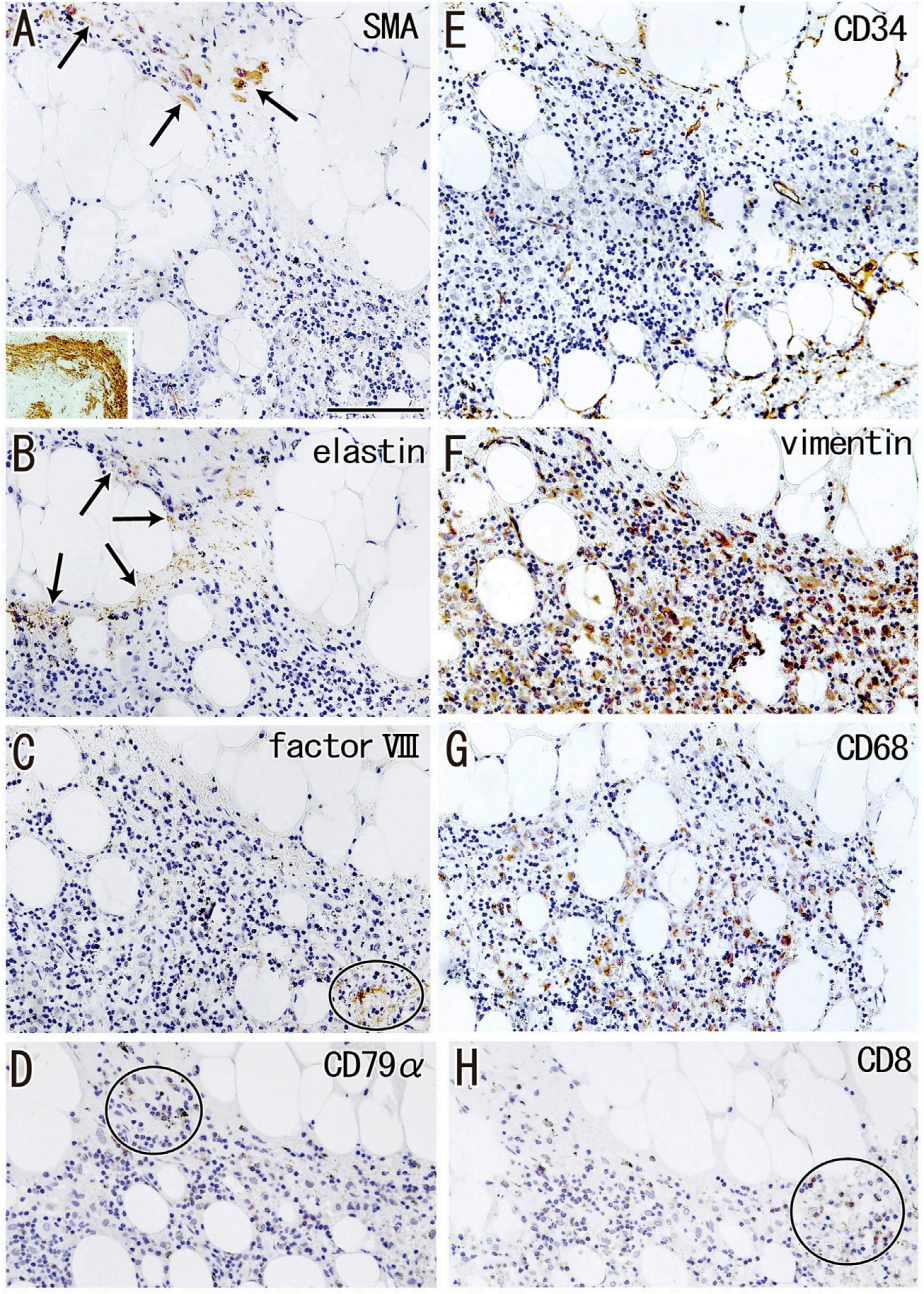
Immunohistochemistry of the fatty degeneration of the medullary sinus. Near sections of Figure 8A and higher magnification views of a site indicated by a circle in Figure 8A (a hilar node removed from a 76-years-old man, non-smoker). The DAB reaction made the positive cells brown. A peninsula-like remnant of the medullary sinus contains smooth muscle actin (SMA)-positive cells [arrows in **(A)]**, elastin-positive fibers [arrows in **(B)**], factor VIII-positive fibers [circle in **(C)**] and CD34-positive vessels **(E)**. Much or less, CD68-positive macrophages **(G)** appear to be positive for vimentin **(F)**. CD79a- and CD8-positve lymphocytes [circle in **(D,H)**] are small in number when compared with abundant lymphocyte-like cells in the remnant medullary sinus. An insert at the lower angle of **(A)** shows a positive control of the present SMA antibody (artery found in the same section). All panels were prepared at the same magnification [scale bar in **(A)**, 0.1 mm].

Fatty tissues (lipomatosis) often extended into the medullary sinuses to replace lymphatic tissue (Figures 1D, 8A), with an incidence >40% in hilar and lower paratracheal nodes (Table 3). This morphology is called fatty degeneration. In fatty tissues, a peninsula- or an island-like remnant of the medullary sinus contained SMA-positive muscle-like cells (Figure 9A), elastin-positive fibers (Figure 9B), factor VIII-positive fibers (Figure 9C) and CD34-positive capillaries (Figure 9E). Many CD68-positive macrophages (Figure 9G) were positive for vimentin (Figure 9F). However, fewer lymphocytes were present (Figures 9D,H) than in usual medullary sinuses (Figures 3A–C). Fatty tissue sometimes invaded into areas of medullary sinus hyalinization, with the former appearing to “eat” the latter collagenous mass (data not shown). Fatty degeneration was not evaluated morphometrically.

### Parameters other than degeneration

**FIGURE 10:**
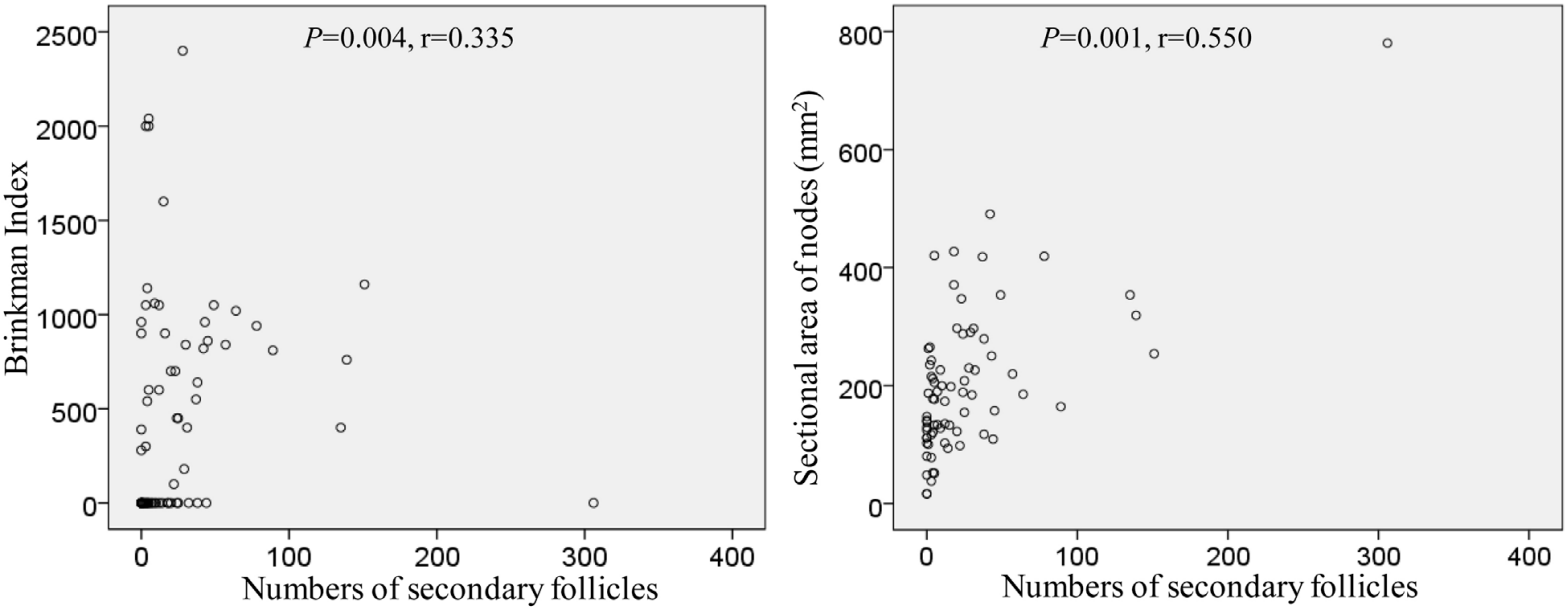
Positive correlation of numbers of secondary follicles with smoking index as well as a sectional area of the node.

The numbers and volume of surgically-removed nodes were quite different between patients. To quantitatively evaluate aforementioned degenerative lesions, we measured 1) a maximum sectional area of the node and 2) numbers of secondary follicles (B lymphocyte clusters with a pale-stained area or germinal center; Figure 3). A total sectional area of multiple nodes at each of five sites (Table 2) ranged from 16.4 to 780.6 mm^2^ (mean ± SD, 202.5 ± 125.4 mm^2^): this value did not correlate with smoking index (*p* = 0.411). Likewise, numbers of secondary follicles per section exhibited a great individual difference: 0-306 (mean ± SD, 26.2 ± 45.8). The greater numbers of secondary follicles were found in patients with higher smoking index (*p* = 0.004, *r* = 0.335). Moreover, a larger node contained greater numbers of secondary follicles (*p* = 0.001, *r* = 0.550): Figure 10 is a graphic demonstration of the positive correlation.

## Discussion

The morphology of lymph node degeneration usually involves a kind of fibrosis, i.e., hyalinization. To our knowledge, however, no comprehensive studies have assessed the correlation of lymph node degeneration with age, immunohistochemical features and the areas of hyalinization within nodes. Moreover, because some of these nodes may have similar onion peel-like features, medullary sinus degeneration may be confused with cortical degeneration. We had hypothesized that early stage morphology would differ between sinus hyalinization and cortical degeneration. The findings of the present study support this hypothesis, in that sinus hyalinization was characterized by factor VIII-positive amorphous eosinophilic matrix, whereas cortical degeneration included follicle-like clusters of small cells other than lymphocytes and macrophages.

Although it was less frequent than sinus hyalinization, cortical degeneration was important for understanding lymph node degeneration because the lesion was likely to occupy almost half of the nodal volume. Benign sinus histiocytosis and multiple clusters of large eosinophilic cells are a common finding of undefined significance in reactive lymph nodes. Pathologically, this finding may be caused by an increase in Langerhans cells (van der Valk and Meijer, 1997). The S100 protein-positive candidates of Langerhans’ cells were actually seen in and around the cortical degeneration, but the positive cells were minority (Figure 7J). Because of its follicle-like shape, cortical degeneration was similar to benign histiocytosis, but it was unlikely to be follicular hyperplasia because of no reactivity of D2-40. Interdigitating cells were also unlikely to exist because of no reactivity of DC-sign (unpublished data). Additional studies are needed to identify the composite cell.

Possibly due to long and deep fixation, D2-40 reactivity seemed to be generally weak in the present histology. Indeed, a positive reaction of follicular dendritic cells was seen in limited specimens. However, lymphatic vessels outside of the node were usually positive. Because hyalinization and other degenerative lesions consistently had no reaction, we did not consider that parts of these lesions were derived from lymphatic vessels.

In the present study, clusters of thick collagenous bundles with low cellularity in the medullary sinuses were regarded as pre-hyalinization, which was seen in many nodes at all sites examined. However, pre-hyalinization could not be easily connected to the early and small hyalinization in the medullary sinuses as the latter were composed of an amorphous matrix rather than fiber bundles. These findings suggest that the early and small lesions of medullary sinus hyalinization were a phase of nodal degeneration different from the advanced lesion containing bulky fiber bundles. Alternatively, these early and small lesions may have resulted from a decrease in arterial blood supply due to arterial sclerosis, as observed for the pathogenesis of pelvic-type hyalinization restricted within a follicle (Taniguchi et al., 2003).

Previous studies described fibrosis in or along the subcapsular sinus (e.g., Hadamitzky et al., 2010; Erofeeva and Mnikhovich 2020). However, to our knowledge, any studies did not identify the elastin-positive nodal capsule. Thus, there seems to be no information as to whether the fibrosis occurs in the capsule or subcapsular sinus. In this context, the present study is the first report of subcapsular sinus hyalinization, which had different immunohistochemical features than medullary sinus hyalinization, although both lesions were fused in advanced cases. In contrast to the usual morphology, the nodal capsules were thicker due to an increase in fibers positive for elastin, factor VIII and/or vimentin in combination with sinus hyalinization. We were unable to clearly identify composite fibers of the newly built layer that seemed to replace the subcapsular sinus, but some of them were SMA-positive. This finding was similar to results showing that areas of follicle-like cortical degeneration were surrounded by SMA-positive fibers. The orbitalis muscle of the human eye has shown the *de novo* development of smooth muscles outside duct-like organs and their transformation to fibrous tissues (Osanai et al., 2011; Cho et al., 2021). Although much less frequent than medullary sinus hyalinization, subcapsular sinus hyalinization seemed to be important for understanding the process of lymph node destruction because the lesion was most likely to stop lymph flow into the node.

Anthracotic macrophages might be believed to induce degenerative changes, such as sinus hyalinization, cortical degeneration and fatty degeneration. Although anthracotic macrophages were most likely to result from smoking, nodes containing few anthracotic macrophages were also likely to carry hyalinization; however, we did not morphometrically evaluate anthracosis. Because few anthracotic macrophages were present in the subcapsular sinus, these macrophages were unlikely to be directly associated with subcapsular sinus hyalinization. We found that neither hyalinization nor cortical degeneration correlated significantly with age or smoking index, and that hyalinization and cortical degeneration were not significantly correlated with each other. Nevertheless, the current correlation was found in a limited population aged 49–82 years. Therefore, we should consider that, until 50-years-old, whether nodal degeneration occurs or not had been determined: this hypothesis was consistent with a major result of Hadamitzky et al. (2010) who showed age-dependent degenerative change of the human inguinal node. Any factors including smoking may cause nodal degeneration during his/her young and middle age. Conversely, after 50 years-old, smoking might not increase nodal degenerative lesions, but it is likely to induce follicular proliferation.

Fatty degeneration or lipomatosis of lymph nodes was quite common, with an incidence as high as 47.8% in hilar nodes. A limited research group seemed to consider lipomatosis as one aspect of the nodal degeneration (e.g., Hadamitzky et al., 2010). Thus, the present Figure 9 might be the first immunohistochemical demonstration showing which medullary cells are persistent in fatty tissues. In fatty tissue, peninsula- and/or island-like remnants of the medullary sinuses contained various cells, including SMA-positive cells similar to vascular smooth muscle cells, as well as elastin- and/or factor VIII-positive fibers derived from the destroyed vessels in the original sinus tissue. CD34-positive vessels also remained in the peninsulas and islands, but they did not contain blood cells. Because the density was lower, the depletion of lymphocytes seemed to be ongoing in the medullary sinus remnants. Overall, during fatty degeneration, the original components of the medullary sinus seemed to decrease in number and volume, while maintaining their original forms.

In the present study, numbers of secondary follicles per section was a limited parameter that significantly correlated with smoking index (*p* = 0.004, *r* = 0.550). Even after 50 years-old, chronic inflammation in the lung with smoking seems to cause the follicular proliferation. Actually, injections of various adjuvants and toxins into the feet have been reported to increase the numbers of follicles and germinal centers in mouse popliteal nodes (Hoshi et al., 1986; Horie and Hoshi, 1989; Horie et al., 1999). However, hyalinization seems not to occur in mice despite that drug-induced fibrosis is known well (Carneiro et al., 2017; Elewa et al., 2018). Conversely, ligation of afferent lymph ducts in mice reduces cellularity in the cortex, but these morphologies differed from the cortical degeneration observed in this study. Decreased lymph inflow in humans, however, was most likely to cause sinus hyalinization and subsequent cortical degeneration. Extensive hyalinization occurred more frequently in proximal (intrapulmonary) nodes than in distal (mediastinal) nodes. Therefore, factors causing degeneration might migrate from proximal to distal along with lymph flow.

Overall, the present study histologically characterized each of morphologies of nodal degeneration in the subcapsular and medullary sinuses as well as in the cortex. Nodal degeneration seemed to interfere with lymph flow through a node to provide a collateral way, resulting in skip-matastasis to a distant node as well as decreased cancer immunity.

## Study limitations

Because the proportional area in the present study was estimated at the maximum sectional area of the node, this study did not include information about the marginal part of the node. The lymph nodes assayed were not metastatic, making immunohistochemistry more informative than anatomical evaluation. However, to evaluate, or even eliminate, the effect of lung cancer, nodes with and without metastasis should be compared (in preparation) although patients with lymph node metastasis is few in number because of the recent early diagnosis of lung cancer. Further studies were also necessary to analyze whether a nodal degeneration is modified by a specific cancer cell type.

## Data Availability

The original contributions presented in the study are included in the article/supplementary material, further inquiries can be directed to the corresponding author.
